# Prediction of conversion to dementia using interpretable machine learning in patients with amnestic mild cognitive impairment

**DOI:** 10.3389/fnagi.2022.898940

**Published:** 2022-08-05

**Authors:** Min Young Chun, Chae Jung Park, Jonghyuk Kim, Jee Hyang Jeong, Hyemin Jang, Kyunga Kim, Sang Won Seo

**Affiliations:** ^1^Department of Neurology, Samsung Medical Center, Sungkyunkwan University School of Medicine, Seoul, South Korea; ^2^Department of Digital Health, Samsung Advanced Institute for Health Sciences and Technology, Sungkyunkwan University, Seoul, South Korea; ^3^Alzheimer’s Disease Convergence Research Center, Samsung Medical Center, Seoul, South Korea; ^4^Department of Neurology, Ewha Womans University Seoul Hospital, Ewha Womans University College of Medicine, Seoul, South Korea; ^5^Biomedical Statistics Center, Data Science Research Institute, Research Institute for Future Medicine, Samsung Medical Center, Seoul, South Korea; ^6^Department of Health Sciences and Technology, Samsung Advanced Institute for Health Sciences and Technology, Sungkyunkwan University, Seoul, South Korea; ^7^Department of Intelligent Precision Healthcare Convergence, Sungkyunkwan University, Suwon, South Korea

**Keywords:** Alzheimer’s disease, amnestic mild cognitive impairment, prediction algorithm, interpretable machine learning, artificial intelligence, clinical decision-support system, SHapley Additive exPlanations (SHAP)

## Abstract

**Purpose:**

Amnestic mild cognitive impairment (aMCI) is a transitional state between normal aging and Alzheimer’s disease (AD). However, not all aMCI patients are observed to convert to AD dementia. Therefore, developing a predictive algorithm for the conversion of aMCI to AD dementia is important. Parametric methods, such as logistic regression, have been developed; however, it is difficult to reflect complex patterns, such as non-linear relationships and interactions between variables. Therefore, this study aimed to improve the predictive power of aMCI patients’ conversion to dementia by using an interpretable machine learning (IML) algorithm and to identify the factors that increase the risk of individual conversion to dementia in each patient.

**Methods:**

We prospectively recruited 705 patients with aMCI who had been followed-up for at least 3 years after undergoing baseline neuropsychological tests at the Samsung Medical Center between 2007 and 2019. We used neuropsychological tests and apolipoprotein E (*APOE*) genotype data to develop a predictive algorithm. The model-building and validation datasets were composed of data of 565 and 140 patients, respectively. For global interpretation, four algorithms (logistic regression, random forest, support vector machine, and extreme gradient boosting) were compared. For local interpretation, individual conditional expectations (ICE) and SHapley Additive exPlanations (SHAP) were used to analyze individual patients.

**Results:**

Among the four algorithms, the extreme gradient boost model showed the best performance, with an area under the receiver operating characteristic curve of 0.852 and an accuracy of 0.807. Variables, such as age, education, the scores of visuospatial and memory domains, the sum of boxes of the Clinical Dementia Rating scale, Mini-Mental State Examination, and *APOE* genotype were important features for creating the algorithm. Through ICE and SHAP analyses, it was also possible to interpret which variables acted as strong factors for each patient.

**Conclusion:**

We were able to propose a predictive algorithm for each aMCI individual’s conversion to dementia using the IML technique. This algorithm is expected to be useful in clinical practice and the research field, as it can suggest conversion with high accuracy and identify the degree of influence of risk factors for each patient.

## Introduction

Amnestic mild cognitive impairment (aMCI) refers to a transitional state between normal aging and dementia (Flicker et al., 1991; Petersen et al., 2001; Sarazin et al., 2007). Previous studies showed that within 3 years, approximately 50% of aMCI patients converted to dementia (Fischer et al., 2007; Espinosa et al., 2013), with an annual conversion rate of 5–25% (Larrieu et al., 2002; Mitchell and Shiri-Feshki, 2009; Alegret et al., 2014). However, some aMCI patients maintain a stable state of cognitive function or reverted to normal cognition (Busse et al., 2006; Mitchell and Shiri-Feshki, 2009). Several factors, including age, sex, neuropsychological test results, and apolipoprotein E (*APOE*) genotype were found to affect the rate of conversion to dementia (Petersen et al., 1995; Daly et al., 2000; DeCarli et al., 2004; Yaffe et al., 2006). Thus, as the clinical outcomes of aMCI patients are heterogeneous, it is important to consider the risk factors of each patient individually while predicting their conversion to dementia.

Several studies have been conducted to create algorithms that predict the conversion of aMCI to dementia (Ravaglia et al., 2006; Tabert et al., 2006; De Simone et al., 2019). Specifically, Jang et al. developed a dementia risk prediction algorithm by using traditional statistical methods, such as multivariate logistic regression (LR) and the nomogram (Jang et al., 2017). However, when the LR is applied to complex multivariate non-linear relationships, it may have low robustness because of the multicollinearity between the variables (Tu, 1996).

Machine learning (ML) techniques, a form of artificial intelligence that is increasingly used in the medical research field, have also been considered in developing prediction algorithms for conversion to dementia (Chen and Herskovits, 2010; Mattila et al., 2012; Hall et al., 2015; So et al., 2017; Zhu et al., 2020; Lian et al., 2021; Qiao et al., 2021). These prediction algorithms are based on computer algorithms that help ML to learn complex relationships with empirical data and to make more accurate decisions (Bishop, 2006; Waljee et al., 2014). Compared to the traditional statistical methods, ML has a lower possibility of overlooking unexpected predictors and potential interactions between variables (Waljee et al., 2014). However, unlike nomograms, ML techniques are not able to show which factors play a major role in the conversion. Thus, interpretable ML (IML) was developed to provide understandable explanations for learning complex outputs with predictive accuracy, descriptive accuracy, and relevancy (Murdoch et al., 2019).

Therefore, in the present study, we aimed to develop an IML algorithm with a higher predictive power than that of LR, which predicts conversion to dementia in aMCI participants in an accurate manner. We used clinical demographics, *APOE* genotype, and neuropsychological results as features that are easily accessible in clinical practice. We also attempted to develop a graphic-based interpretable method to show which risk factors influence conversion to dementia, and to what extent, in individual aMCI participants.

## Materials and methods

### Participants

We conducted a cohort study among participants with aMCI who visited the Samsung Medical Center (SMC) in South Korea from June 2007 to December 2019 and were followed-up for at least 3 years after baseline neuropsychological tests. In total, 705 participants with aMCI were enrolled in this study. All aMCI subjects met the following criteria for aMCI (Albert et al., 2011): (1) subjective memory complaints by participants or caregivers; (2) objective memory decline below –1.0, standard deviation (SD) on either verbal or visual memory tests; (3) normal activities of daily living (ADL), as judged clinically; and (4) not demented.

All the subjects underwent neurological examination, laboratory tests, including *APOE* genotype, and neuropsychological tests. We excluded participants with secondary causes of cognitive impairment through laboratory tests, such as vitamin B_12_/folate determination, syphilis serology, and thyroid function tests. In addition, participants with structural lesions, such as territorial infarction, intracranial hemorrhage, brain tumor, traumatic brain injury, hydrocephalus, or severe white matter hyperintensities on brain magnetic resonance imaging (MRI), were excluded.

The study was approved by the Institutional Review Board of SMC, and informed consent was obtained from all participants and caregivers.

### Neuropsychological assessments

All the participants underwent the Seoul Neuropsychological Screening Battery (SNSB), a standardized neuropsychological battery widely used in South Korea (Kang and Na, 2003; Kang et al., 2016). Four major cognitive domains were evaluated: memory, language, visuospatial, and frontal/executive function. If the z-score of SNSB was below −1.0 SD of age and education, it was considered impaired.

The scorable tests are comprised of the Korean version of the Boston Naming Test (Kim and Na, 1999), Rey-Osterrieth Complex Figure Test (RCFT) (Kang and Na, 2003), which involves copying, immediate and 20-min delayed recall, and recognition, the Seoul Verbal Learning Test (SVLT) (Kang and Na, 2003), which includes three learning-free recall trials of 12 words, a 20-min delayed recall trial of these 12 items, and a recognition test, the contrasting program (instructing the patient to raise the second and third fingers when the examiner raises the second finger, and to raise the second finger when the examiner raises the second and third fingers), go/no-go test (changing the initial rule as follows: instructing the patient to make a fist in respond to examiner’s raising the second and third fingers) (Dubois et al., 2000), and phonemic and semantic Controlled Oral Word Association Tests (COWAT) (Kang et al., 2000). In addition, the ideomotor praxis and the total calculation score were evaluated. The Korean version of the Mini-Mental State Examination (K-MMSE) and clinical dementia rating-sum of boxes (CDR-SOB) of all the participants were investigated (Kang et al., 2016).

### Follow-up

All the participants underwent two or more SNSB during a follow-up period of at least 3 years. Dementia was diagnosed on the basis of the criteria of the fourth edition of the Diagnostic and Statistical Manual of Mental Disorders and required evidence of cognitive deficits (confirmed by neuropsychological testing) and social and/or occupational dysfunction (confirmed by ADL impairment). The criteria of the National Institute of Neurological and Communicative Disorders and Stroke and the Alzheimer’s Disease and Related Disorders Association were used for the diagnosis of probable AD (McKhann et al., 2011). A consensus panel and an experienced neurologist reviewed the interview records and neuropsychological results of each aMCI patient and confirmed the conversion to dementia in the SMC cohort.

The primary outcome was defined as conversion to dementia within 3 years of the baseline neuropsychological test. The predictive algorithm used variables, such as age, gender, years of education, neuropsychological features, *APOE* ε2, and *APOE* ε4 status as the potential predictors.

### Feature selection

Three major steps were performed to select variables: First, domain knowledge was used to remove the unnecessary variables from the results of neuropsychological tests; second, the remaining variables were used to confirm the significance of the variables through LR analysis for a single variable and remove the insignificant variables; and third, one of the variables suspected of multicollinearity was removed or integrated through the correlation coefficient. We specified the primary outcome as 3-year dementia conversion and included features, such as demographics, *APOE* genotypes, and neuropsychological features (including K-MMSE and CDR-SOB) selected using the above process. The selected features were used as inputs for predictive model building, and as potential predictors for model interpretation.

### Algorithm constructions

Eighty percent of the total data was randomly selected by the matching class imbalance and used it to develop the predictive algorithm, and the remaining 20% was used for the algorithm test. Stratified 5-fold cross-validation was repeated five times by random dataset splitting, and Bayesian optimization was used for hyperparameter tuning. Five types of ML models were developed: multivariable LR, random forest (RF), support vector machine (SVM), artificial neural network (ANN) and extreme gradient boost (XGB).

### Statistical analyses

The performance of the model was compared by using areas under the receiver operating characteristic curve (AUCs) with DeLong test (*P*-value < 0.05 indicated statistical significance) (DeLong et al., 1988). Statistical analyses were performed using the Daim (v1.1.0) package in R 4.1.2 (R Core Team, 2021).

### Interpretation methods

The interpretation of the developed ML models was based on both global and local perspectives. IML analysis was carried out using R 4.1.2 (R Core Team, 2021), the caret (v6.0-90), the iml (v0.10.1), the vip (v0.3.2), the pdp (v0.7.0), the breakDown (v0.2.1), SHAPforxgboost (v0.1.1), the caret (v6.0-90), the DALEX (v2.3.0), and the modelStudio (v3.0.0) packages.

#### Global interpretation

The global analysis method was used to evaluate the overall performance of the developed model, which we evaluated through the model performance, feature importance (Breiman, 2001; Fisher et al., 2019), and partial dependence (Friedman, 2001). The ML model performance of the four groups divided by gender and age was measured by accuracy and AUC. The feature importance is to observe a lowered performance change by randomly mixing a specific feature. The partial dependence plot (PDP) is a global interpretation method in the ML model that shows the marginal effect of one or two features on the prediction result (Friedman, 2001).

#### Local interpretation

The local analysis method interpreted the prediction results for individual participants. In this study, we implemented Individual Conditional Expectations (ICE) (Goldstein et al., 2015), Break-down (Robnik-Šikonja and Kononenko, 2008), and SHapley Additive exPlanations (SHAP) (Lundberg and Lee, 2017). First, ICE (or Ceteris-paribus) plots display one line per individual that shows how the individual’s prediction changes when a feature changes (Goldstein et al., 2015). Other feature values are fixed with the individual’s data. Second, Break-down plots show feature attributions; that is, the prediction is decomposed into contributions that can be attributed to different interpretive features (Robnik-Šikonja and Kononenko, 2008). A plot can be drawn by adding or subtracting each feature contribution one by one on the basis of the average predicted value for all datasets. Finally, SHAP explains individual predictions by computing the contribution of each feature to the prediction. This is based on the game theoretically optimal Shapley values (Lundberg and Lee, 2017). Unlike break-down plots, the order of adding features is calculated by numerous trials; therefore, the mean and SD is estimated.

We plotted three local interpretations above with the XGB model using six exemplary patients. Supplementary Table 1 shows demographic and dementia conversion information. Also, we collected all IML results and developed dashboards with a graphical view of each patient’s analysis results.

## Results

### Demographics and clinical characteristics

Table 1 shows the patient demographics and clinical characteristics. The model-building and validation datasets were composed of 565 and 140 participants, respectively. Among the aMCI participants of the development set, 36.1% (204/565) of the participants were observed to convert to dementia within 3 years. In the validation set, 50 out of 140 participants (35.7%) converted to dementia, which is similar to the conversion rate in the development set. Among participants who converted to dementia, 90.2% (*n* = 229) progressed to clinical AD–type dementia by meeting the core clinical criteria for probable AD (McKhann et al., 2011), and 9.8% to other types of dementia including subcortical vascular dementia (*n* = 12, 4.7%), frontotemporal dementia (*n* = 2, 0.8%), dementia with Lewy bodies (*n* = 2, 0.8%), and others (*n* = 9, 3.5%).

**TABLE 1:** Demographics of the study.

Feature	Training set (*N* = 565)		Validation set (*N* = 140)	
	Mean	SD (%)	Mean	SD (%)
Conversion to dementia	204	(36.1%)	50	(35.7%)
Age (years)	71.6	7.8	72.2	7.6
Sex – Women	348	(61.6%)	84	(60.0%)
Education (years)	11.1	5.2	11.1	4.8
*APOE* ε4 carrier	214	(37.9%)	45	(32.1%)
*APOE* ε2 carrier	46	(8.1%)	9	(6.4%)
K-BNT	39.9	10.1	39.6	10.3
Ideomotor praxis	4.2	1.2	4.2	1.2
Calculation total score	10.9	2.0	10.6	2.1
RCFT copy score	29.7	6.3	29.7	5.7
RCFT copy time (seconds)	258.5	124.3	273.5	139.4
SVLT delayed recall	2.6	2.5	2.5	2.4
SVLT recognition score	18.3	2.8	18.4	2.4
RCFT delayed recall	6.9	5.4	6.8	4.8
RCFT recognition score	18.2	2.3	18.3	2.3
Contrasting program	19.1	2.8	19.0	2.9
Go/no-go	16.9	5.0	16.8	4.9
COWAT animal	12.5	4.2	12.6	4.3
K-MMSE	25.9	3.2	25.6	3.2
CDR-SOB	1.5	0.9	1.5	0.9

The numbers are mean and standard deviation (or percentage in parenthesis) of the training and validation sets.

APOE, apolipoprotein E; K-BNT, Korean version of the Boston Naming Test; RCFT, Rey–Osterrieth Complex Figure Test; SVLT, Seoul Verbal Learning Test; COWAT, Controlled Oral Word Association; K-MMSE, Korean version of the Mini-Mental State Examination; SD, standard deviation; CDR-SOB, clinical dementia rating-sum of boxes.

The following 19 features were used for model building: age, gender, education, *APOE* ε2, *APOE* ε4, K-BNT, ideomotor apraxia, calculation total score, RCFT copy score, RCFT copy time, SVLT delayed recall, SVLT recognition score, RCFT delayed recall, RCFT recognition score, contrasting program, go/no-go test, COWAT animal, K-MMSE, and CDR-SOB.

### Global interpretation

The global interpretation results on the three methods are as follows:

#### Algorithm performance

**FIGURE 1:**
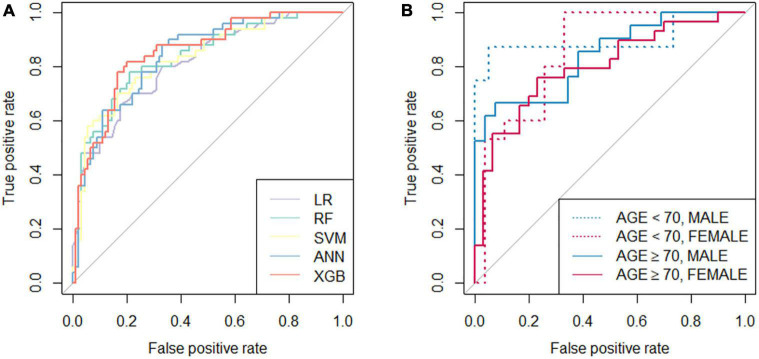
Receiver operation characteristic (ROC) curves of the classifiers. **(A)** ROC curves of five developed classifiers; **(B)** ROC curves of the extreme gradient boost classifier tested with validation set divided by age (threshold of 70 years old) and gender. LR, logistic regression; RF, random forest; SVM, support vector machine; ANN, artificial neural network; XGB, extreme gradient boost.

The performance of the developed classifiers on validation set and the optimized hyperparameters is shown in Table 2. The XGB model showed the highest performance (accuracy 0.807, AUC 0.852) compared to the other models. Figure 1A shows the receiver operating characteristic curve of the developed classifiers. Statistical tests showed that the AUCs of the XGB and the LR models were significantly different (*P-*value < 0.05). The hyperparameters of best performed XGB model was as follows: booster = gbtree, eta = 0.1, max_depth = 6, min_child_weight = 17, subsample = 0.81, colsample_bytree = 0.66. The hyperparameters of other models were as follows: mtry = 4 for RF, sigma = 0.020 and *C* = 0.849 for SVM, and size = 4 and decay = 0.32 for ANN. We determined the XGB to be the best-performing classifer and proceeded with the model interpretation. Also, we divided test set into 4 groups by gender and age: (1) age < 70 and male (*n* = 20), (2) age < 70 and female (*n* = 29), (3) age ≥ 70 and male (*n* = 36), (4) age ≥ 70 and female (*n* = 55). The prediction result from XGB model of each group was (1) 0.902, (2) 0.838, (3) 0.865, and (4) 0.828, respectively (Figure 1B).

**TABLE 2:** Performance of classifiers on validation set.

Classifier	Accuracy	AUC
Logistic regression	0.743	0.813
Random forest	0.771	0.834
Support vector machine	0.800	0.830
Artificial neural network	0.757	0.841
Extreme gradient boost	0.807	0.852

Each classifier’s accuracy, area under the receiver operating characteristic curve, and optimized hyperparameters as presented.

AUC, area under the receiver operating characteristic curve.

#### Feature importance

**FIGURE 2:**
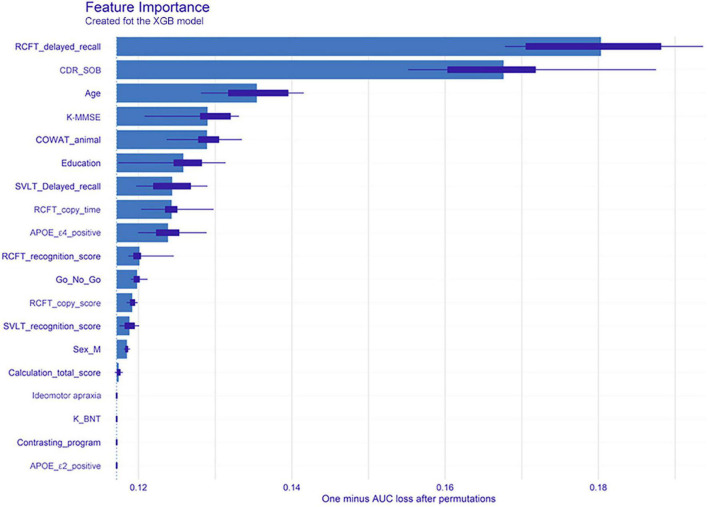
Feature importance of the extreme gradient boost model. The bars indicate the feature importance, while the interval bands indicate difference due to random permutations. From the model, clinical neuropsychological features of RCFT delayed recall, clinical dementia rating-sum of boxes, and age were noted as important factors to the global performance. XGB, extreme gradient boost; RCFT, Rey–Osterrieth Complex Figure Test; CDR-SOB, clinical dementia rating-sum of boxes; K-MMSE, Korean version of the Mini-Mental State Examination; COWAT, Controlled Oral Word Association; SVLT, Seoul Verbal Learning Test; APOE, apolipoprotein E; K-BNT, Korean version of the Boston Naming Test; AUC, area under the receiver operating characteristic curve.

Figure 2 shows feature importance of XGB, where the bars indicate feature importance, and the interval bands indicate difference due to random permutations. According to the result, clinical neuropsychological features of RCFT, CDR-SOB, as well as age were important factors to the global performance.

#### Partial independence

**FIGURE 3:**
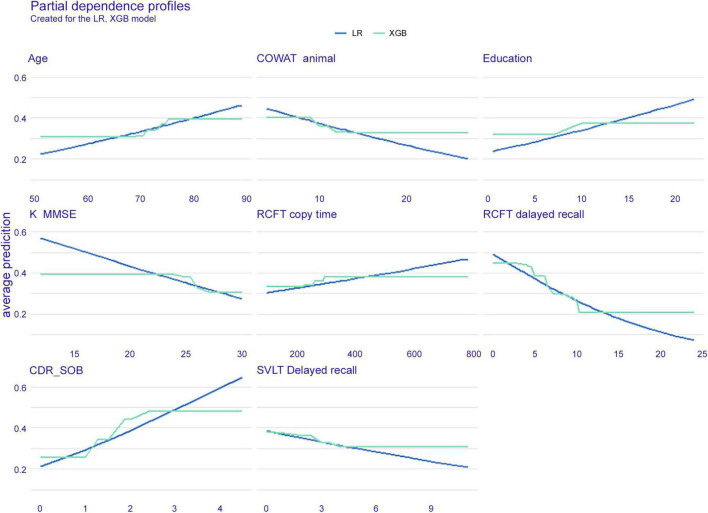
Partial dependence plot of six features. The extreme gradient boost (blue) model and logistic regression (green) model are presented. LR, logistic regression; XGB, extreme gradient boost; COWAT, Controlled Oral Word Association; K-MMSE, Korean version of the Mini-Mental State Examination; RCFT, Rey–Osterrieth Complex Figure Test; CDR-SOB, clinical dementia rating-sum of boxes; SVLT, Seoul Verbal Learning Test.

In Figure 3, the PDP of six features is shown with the XGB and LR models. It can be explained that under the condition that other features are fixed, the possibility of dementia conversion increases with age, while it decreases when the RCFT delayed recall score increases. The slope patterns of the XGB and LR were similar.

### Local interpretation

The local interpretation results on three methods are as follows.

#### Individual conditional expectations

**FIGURE 4:**
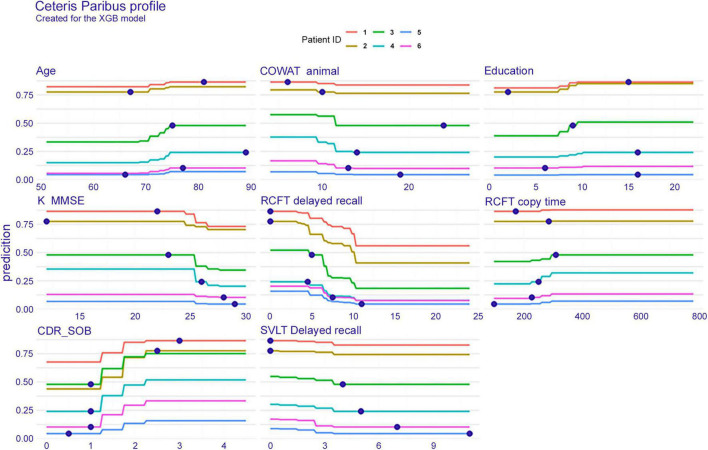
Individual conditional expectation on eight features when predicted with the extreme gradient boost model. The results of a total of six patients are plotted in different line colors. XGB, extreme gradient boost; COWAT, Controlled Oral Word Association; K-MMSE, Korean version of the Mini-Mental State Examination; RCFT, Rey–Osterrieth Complex Figure Test; CDR-SOB, clinical dementia rating-sum of boxes; SVLT, Seoul Verbal Learning Test.

Figure 4 shows the ICE plot, which presents eight features for six individuals. To explain the result on patient number 3 (green line), the probability of dementia conversion increases between the ages of 70 and 75 years. The age of this patient is 75 years as seen in a blue dot on the green line, the interpretation plot shows the prediction value (*y*-axis), that is, the conversion probability, indicating approximately 0.5 within 3 years. Likewise, regarding RCFT delayed recall, this subject scored 5; therefore, the conversion possibility was approximately 0.5. If the patient had performed the test better and obtained a higher score, the conversion probability would be reduced.

#### Break-down plots

**FIGURE 5:**
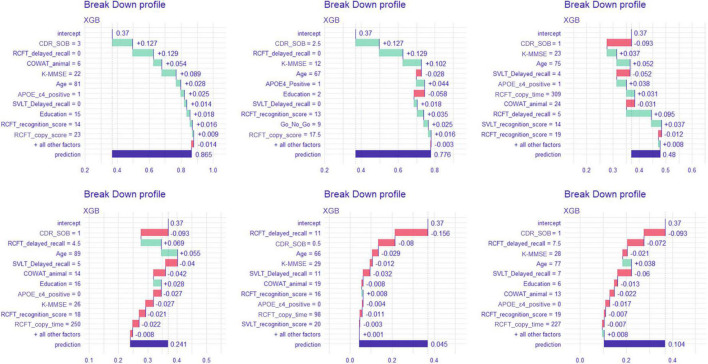
Break-down plot on six patients when predicted with the extreme gradient boost model. XGB, extreme gradient boost; CDR-SOB, clinical dementia rating-sum of boxes; RCFT, Rey–Osterrieth Complex Figure Test; K-MMSE, Korean version of the Mini-Mental State Examination; COWAT, Controlled Oral Word Association; SVLT, Seoul Verbal Learning Test; APOE, apolipoprotein E.

Figure 5 shows the break-down plots in six individuals, with the XGB model. In patient number 1, the most upper left plot, the subject had a sum of box value of 3, which attributes as much as 0.127 to the baseline mean prediction value of 0.36. In the same way, the RCFT delayed recall value of 0 contributes as much as 0.127 to the prediction.

#### SHapley Additive exPlanations

**FIGURE 6:**
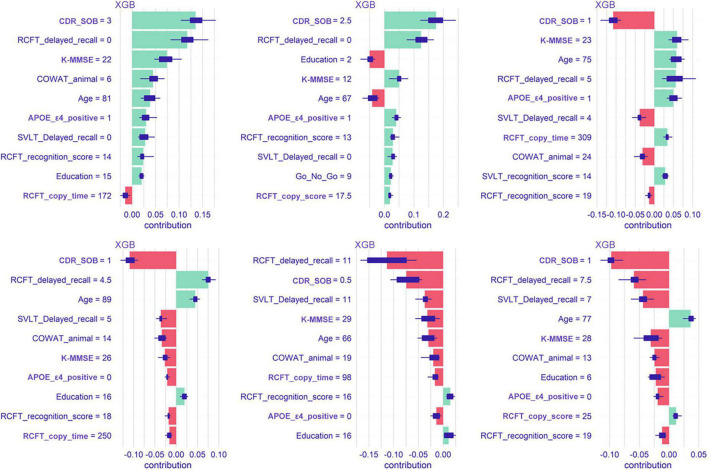
Shapley values plot of six patients when predicted with the extreme gradient boost model. XGB, extreme gradient boost; CDR-SOB, clinical dementia rating-sum of boxes; RCFT, Rey–Osterrieth Complex Figure Test; K-MMSE, Korean version of the Mini-Mental State Examination; COWAT, Controlled Oral Word Association; SVLT, Seoul Verbal Learning Test; APOE, apolipoprotein E.

Figure 6 shows Shapley values plot of six individuals. In patient number 1 (the most upper left plot), the feature that contributed the most to predicting dementia conversion is the CDR-SOB. In patient number 5 (lower middle plot), RCFT delayed recall contributed most to the conversion.

### Graphic-based overall interpretation on individuals

**FIGURE 7:**
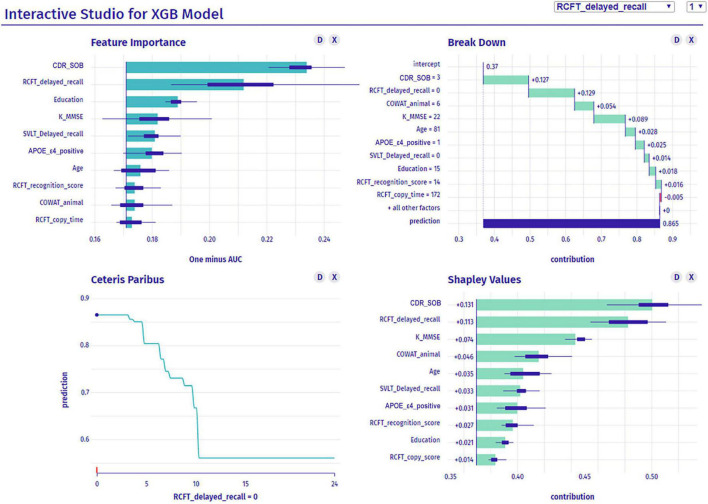
Dashboard for a patient’s interpretation for predicting dementia conversion. XGB, extreme gradient boost; CDR-SOB, clinical dementia rating-sum of boxes; RCFT, Rey–Osterrieth Complex Figure Test; K-MMSE, Korean version of the Mini-Mental State Examination; SVLT, Seoul Verbal Learning Test; APOE, apolipoprotein E; COWAT, Controlled Oral Word Association.

Figure 7 shows the dashboard displaying the global and the local interpretation of patient 1. We collected all the IML results above and developed a dashboard that provides a graphical view of each patient’s analysis results by displaying them on a screen (Figure 7). It not only provides the probability of aMCI to dementia conversion, but also presents quantitative information on the risk factors attributed to the conversion.

## Discussion

In the present study, using the clinical and neuropsychological features of carefully phenotyped aMCI patients, we developed an algorithm to predict conversion to dementia by applying the IML technique. Our major findings are as follows. First, among the ML techniques, the XGB model showed the best accuracy, which was superior to that of LR. Second, variables, such as visual memory delayed recall, CDR-SOB, age, K-MMSE score, frontal executive function, education, verbal memory delayed recall, visuospatial function, and *APOE* genotype were important features for creating the algorithm. Finally, ICE and SHAP analyses allowed for the interpretation of variables acted as important factors in the conversion to dementia of each aMCI patient. Taken together, our findings suggest that an algorithm using the IML technique enables us to individually predict the conversion of patients with aMCI to dementia within 3 years in clinical practice and the research field. Using our newly developed IML algorithm, we predict that, with the aid of visualized graphs, patients will be able to more easily understand the neuropsychological factors that are at risk, which would become a further step toward precision medicine.

In the present study, when compared with other algorithms including LR, the XGB model showed the best performance with an AUC of 0.852 and an accuracy of 0.807. Thus, these findings suggest that our newly developed algorithm with the XGB model overcomes this limitation and results in better AUC and accuracy than LR. If the predictive algorithm is applied to the electronic medical record system, the conversion rate would be readily calculated in clinical practice with more accuracy.

The second major finding was that RCFT delayed recall, CDR-SOB, age, K-MMSE, COWAT-animal, education, SVLT delayed recall, RCFT copy time, and *APOE* genotype were the important factors in the IML algorithm, which is consistent with previous studies. Consistent with our findings, MMSE (Hou et al., 2019), CDR-SOB (Daly et al., 2000; Dickerson et al., 2007; Montano et al., 2013; Woolf et al., 2016), and frontal/executive dysfunction, which can be examined by the COWAT-animal test (Lezak et al., 2004), were found to be the predictors of conversion to dementia in other studies (Tabert et al., 2006; Jung et al., 2020). The *APOE* ε4 genotype was also found to play an important role in conversion to dementia, which was again consistent with previous studies (Petersen et al., 1995; Mosconi et al., 2004; Elias-Sonnenschein et al., 2011).

In our previous studies (Ye et al., 2015; Jang et al., 2017), the odds ratio of conversion to dementia was higher in Verbal-aMCI patients than in Visual-aMCI patients. However, our global interpretation results showed that the RCFT delayed recall score (visual memory) had higher feature importance than the SVLT delayed recall score (verbal memory), which is thought to be due to differences in the classification of participants. The previous studies defined Visual-aMCI as only visual memory impairment, Verbal-aMCI as only verbal memory impairment, and Both-aMCI as visual and verbal memory impairment, and then analyzed the odds ratio compared to Visual-aMCI. On the other hand, we analyzed the variables of the RCFT delayed recall score and SVLT delayed recall score together with other neuropsychological test scores of all participants without classification.

There are also some debates on the educational effects in participants with aMCI among studies. Specifically, a previous study (Cooper et al., 2015) did not show that high educational levels predict conversion to dementia in participants with aMCI. However, another study from our group showed that highly educated aMCI participants were at a higher risk of conversion to AD dementia than less educated aMCI participants (Ye et al., 2013). Furthermore, early stage aMCI participants with higher levels of education showed a slower cognitive decline while late-stage aMCI participants with higher levels of education showed a more rapid cognitive decline. Thus, our present findings that aMCI patients with higher education levels were more likely to convert to dementia should be replicated in the future studies with larger MCI participants.

Some studies have proposed an algorithm for differentiating cognitive decline using ML methods, including the Disease State Index, naïve Bayes, Bayesian network classifier with inverse tree structure, decision tree, SVM, multiple-layer perceptrons, Begging, RF, and rule-based classifier (Chen and Herskovits, 2010; Hall et al., 2015; So et al., 2017; Bansal et al., 2018; Bhagyashree et al., 2018; Zhu et al., 2020). Beheshti et al. also developed a predictive algorithm with feature ranking and a genetic algorithm, which can predict the conversion rate to dementia after 3 years (Beheshti et al., 2017). However, compared to previous studies, the present study is meaningful in that we predicted the conversion of aMCI to dementia with IML, especially by presenting the attribution of each feature to the prediction. Thus, the IML predictive algorithm used in our study might be more useful in clinical practice because it is composed of clinical data that are widely and commonly used for evaluating cognition status.

Our final major finding was that our IML, which consisted of the ICE and SHAP analyses, allowed for the interpretation of variables that acted as important factors in the conversion to dementia in each patient. Therefore, we suggest that our IML is an improved predictive algorithm that has both the high accuracy of ML and the advantage of the nomogram. Identifying the specific factors that influence conversion to dementia for each aMCI patient will be helpful for the development of personalized intervention strategies in the future.

To our knowledge, our study is the first to develop an IML algorithm to predict conversion to dementia within a large sample size of well-phenotyped aMCI patients. Another strength of this study is that the IML algorithm was based on variables that are most commonly used in clinical practice, specifically neuropsychological test results and *APOE* genotype. However, this study has some limitations. First, MRI volumetry and cortical thickness, which are highly correlated with neurodegenerative dementia, were not used in this algorithm. Future studies incorporating structural brain MRI information are required to achieve higher predictive power. Second, since we did not perform amyloid and tau positron emission tomography in all participants, we could not determine the biomarker guided diagnosis in our participants. Third, the number of samples to train the model might not be large enough because of the limited number of subjects of 3-year followed-up. Finally, since this study was conducted only at SMC, there is a limitation regarding the generalizability of the outcomes. External validation in an independent cohort should be conducted in the future. Nevertheless, our study is noteworthy in demonstrating that the IML algorithm is able to estimate the individual risk of conversion to dementia in each aMCI patient.

## Conclusion

This study was able to develop an IML algorithm to predict conversion to dementia in aMCI patients. This IML algorithm is expected to be useful in clinical practice and the research field as it can identify the degree to which individual risk factors influence each patient.

## Data availability statement

The raw data supporting the conclusions of this article will be made available by the authors, without undue reservation.

## Ethics statement

The studies involving human participants were reviewed and approved by the Institutional Review Board of Samsung Medical Center. The patients/participants provided their written informed consent to participate in this study.

## Author contributions

MC, CP, and JK: conceptualization and formal analysis and investigation. CP and JK: methodology. MC and CP: writing – original draft preparation. JJ, HJ, KK, and SS: writing – review and editing. SS: funding acquisition. KK and SS: supervision. All authors contributed to manuscript revision, read, and approved the submitted version.
