# Supplementary material for: Prediction of conversion to dementia using interpretable machine learning in patients with amnestic mild cognitive impairment
Source: Front Aging Neurosci. 2022 Aug 5;14:898940. doi: 10.3389/fnagi.2022.898940 (PMC9389270; doi:10.3389/fnagi.2022.898940)
Supplement: Supplementary file 1 [file Table_1.DOCX]

**SUPPLEMENTARY** **Table 1 |** Demographics of six patients for local interpretation.

| **No.** | **Age** | **Sex** | **Education** | ***APOE* genotype** | **Conversion** |
| --- | --- | --- | --- | --- | --- |
| 1 | 81 | Woman | 15 | ε4/ε4 | Yes |
| 2 | 67 | Woman | 2 | ε4/ε4 | Yes |
| 3 | 75 | Man | 9 | ε3/ε4 | Yes |
| 4 | 89 | Man | 16 | ε2/ε3 | No |
| 5 | 66 | Man | 16 | ε3/ε3 | No |
| 6 | 77 | Woman | 6 | ε3/ε3 | No |

*Abbreviations: APOE, apolipoprotein E.*
